# Pre-organized landscape of T cell surface

**DOI:** 10.3389/fimmu.2023.1264721

**Published:** 2023-09-13

**Authors:** Yunmin Jung

**Affiliations:** ^1^ Department of Nano-Biomedical Engineering, Advanced Science Institute, Yonsei University, Seoul, Republic of Korea; ^2^ Center for Nanomedicine, Institute for Basic Science, Seoul, Republic of Korea

**Keywords:** pre-organization, pre-cluster, preassembly, T cell membrane, microvilli, T cell receptor, CD45

## Abstract

T cell activation is initiated by the recognition of specific antigenic peptides and subsequently accomplished by complex signaling cascades. These aspects have been extensively studied for decades as pivotal factors in the establishment of adaptive immunity. However, how receptors or signaling molecules are organized in the resting state prior to encountering antigens has received less attention. Recent advancements in super-resolution microscopy techniques have revealed topographically controlled pre-formed organization of key molecules involved in antigen recognition and signal transduction on microvillar projections of T cells before activation and substantial effort has been dedicated to characterizing the topological structure of resting T cells over the past decade. This review will summarize our current understanding of how key surface receptors are pre-organized on the T-cell plasma membrane and discuss the potential role of these receptors, which are preassembled prior to ligand binding in the early activation events of T cells.

## Introduction

T cells play a major role in establishing and orchestrating adaptive immune responses that defend the host against viral infections, cancer, and autoimmune diseases. Triggering of the antigen-specific T cell receptor (TCR) and subsequent signaling cascades have been extensively reviewed elsewhere (1, 2). This review focuses on the pre-organization of the T cell plasma membrane in the quiescent state, *i.e*., prior to antigen engagement, in order to provide a more comprehensive view of the molecular organization involved in the optimization of T cell activity. Pre-organized receptors are often reported to form nanoscale clusters. Although investigations using immunogold electron microscopy (EM) have revealed some pre-clustered receptors, many pre-organized proteins have remained unresolved due to diffraction limit until the advent of super-resolution imaging. Super-resolution imaging enables to resolve sub-diffraction limit by utilizing single-molecule localization precision (photoactivated localization microscopy (PALM) (3, 4), stochastic optical reconstruction microscopy (STORM) (5), points accumulation for imaging in nanoscale topography (PAINT) (6)*, etc.*), or by shaping or modulating the illumination (stimulated emission depletion (STED) (7), structured-illumination microscopy (SIM) (8), lattice light sheet microscopy (LLSM (9)*, etc.*). Another approach for super-resolution imaging, expansion microscopy (ExM) technique, improves resolution by physical expansion of hydrogel-embedded specimens (10). The detailed technical aspects of the super-resolution imaging techniques that are used for studying pre-organized clusters are reviewed elsewhere (11–13).

Receptor pre-organization is established and maintained in the naïve or resting T cell state and is, therefore, more likely to be controlled by the intrinsic physical properties of the plasma membrane or protein itself, rather than by the outcome of specific signaling events triggered by receptor-ligand interactions. For instance, the heterogeneous lipid composition, especially the presence of cholesterol or sphingolipids, leads to the compartmentalization of lipid domains known as lipid rafts within the plasma membrane. Lipid rafts are small (10–200) and dynamic assemblies capable of being clustered into microscopic domains (>300 nm) by protein–protein or protein–lipid interactions (14–16). These domains are commonly characterized by their liquid-ordered (Lo) phase (17, 18). The preferential associations between cholesterol and saturated lipids drive the formation of denser and thicker membrane domains that selectively recruit certain lipids and proteins, thereby contributing to receptor clustering, protein sorting, and downstream signaling events (15, 19, 20). Multimeric interactions between receptors can drive the formation of nanodomains in the plasma membrane (21, 22). Topographical structures, such as protrusions, can also cause the segregation of receptors (23–25). The ability of membrane proteins to associate with cortical cytoskeleton may also affect the localization and clustering of receptors (24, 26, 27). Presumably, the combination of these properties influences the arrangement of a pre-organized landscape on the T-cell plasma membrane, making it ready for sensitive, selective, and effective triggering and activation when they encounter cognate antigens.

## Pre-existing clusters of T cell receptors

The TCR complex is composed of a disulfide-linked dimers consisting of TCRαβ, CD3γϵ and CD3δϵ, and a CD3ζζ homodimer (28). The extracellular domain of the TCRαβ dimer binds to specific antigens presented on MHC molecules on antigen presenting cells (APCs), leading to initiation of TCR signaling. This signaling is initiated by phosphorylation of multiple immune receptor tyrosine-based activation motifs (ITAMs) located in the intracellular domains of the CD3 subunits (1, 29, 30). Rapid changes in TCR localization have been observed upon activation using conventional microscopy. When TCRs bind to cognate antigens, they accumulate in the central region of the interface between the T cell and APC, called the immunological synapse (IS), forming clusters 1-5 µm in diameter observed by confocal microscopy (31, 32). Total internal reflection fluorescence microscopy (TIRFM) has been used to visualize TCR clusters of 300-800 nm in diameter along with other membrane associated signaling molecules, which formed within seconds after contact with antigen-functionalized glass surfaces or synthetic planar lipid bilayers (33–36). With the use of these conventional microscopies, TCRs appeared to be randomly distributed before activation. However, some studies have suggested that at least a fraction of TCRs forms dimers or multimers on unstimulated T cells (37–40), while other studies have suggested that TCRs are predominantly monovalent (41–44).

**Figure 1:**
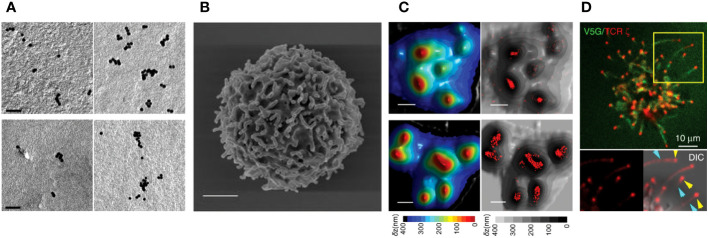
TCR pre-clusters of on human T cells. **(A)** Pre-clusters of TCRs on 2D surface of T cell membrane. Immunogold-EM images showing the distribution of gold particles of anti-CD3ϵ in human resting (upper-left) and effector (upper-right) T cells and anti-TCRβ in human resting (lower-left) and effector (lower right) T cells. (Scale bars: 50 nm). Reproduced from Kumar et al. (45) with permission from the Journal. **(B)** A scanning electron microscopy image of a human resting T cells isolated from peripheral blood mononuclear cells showing microvilli protrusions on the surface (Scale bar: 1 µm) (24). **(C)** High accumulation of TCRs on microvilli of human T cells. 3D surface topography reconstruction maps obtained from VA-TIRFM measurements of resting (upper-left) and effector (lower-left) T cells and STORM images of αβTCR (upper-right) and CD3ϵ molecules (lower-right) of the cells overlaid with the 3D surface reconstruction map presented on the left. Color bars indicate the relative distance (δz) from the surface. (Scale bars: 500 nm). **(B**, **C)** are reproduced from Jung et al. (24) with Copyright 2016 National Academy of Sciences. **(D)** TCRs localized at the tips of microvilli of Jurkat T cells. Airyscan confocal image of activated Jurkat T cell expressing V5G-GFP (green) and TCRζ-ptdTomato (red). Reproduced from Kim et al. (46), licensed under Creative Commons (CC BY 4.0).

Immunogold-EM has been used to directly visualize multimeric TCRs on unstimulated cells (40, 45) (**Figure 1A**). After the introduction of fluorescence super-resolution imaging, Davis et al. reported the existence of pre-formed TCR nanoclusters on the plasma membrane of resting T cells using fluorescence super-resolution microscopic technique, PALM or direct STORM (*d*STORM) (47, 48). TCR pre-clusters (protein islands) with a radius of 35–70 nm are observed in quiescent mouse T cells bound to both non-activating and activating surfaces. These pre-clusters then come together to form microclusters upon activation of the T cells.

The presence of TCR pre-clusters was confirmed by transmission EM (TEM) imaging, where they appeared as clusters having a 40-300 nm radius on resting T cells. Smaller pre-existing TCR nanoclusters have also been reported on T cells spread on non-activating surface using PALM or *d*STORM (49, 50). In addition, nano-sized TCR clusters have been observed on lymph node-resident T cells *in vivo* using *d*STORM and SIM (51). These studies revealed the existence of nanoscale pre-clusters prior to activation, but information on the three-dimensional (3D) distribution of TCRs on resting T cells was missing as the T cell plasma membrane is altered when the cells are spread on the two-dimensional (2D) surface.

In 2016, we first reported the localization of TCRs in relation to native surface protrusions using a combination of variable angle of TIRF microscopy (VA-TIRFM) and *d*STORM (24). Our study revealed that CD3ϵ and TCRαβ are highly enriched on the microvilli of freshly isolated or effector human T cells in their quiescent state (**Figures 1B, C**). Microvilli are thin (~70-200 nm in diameter) and short (0.3-0.5µm in median length) actin-based finger-like membrane protrusions that are a dominant feature of the T cell surface (2-4 microvilli/μm^2^) (52, 53) (**Figure 1B**). A study using ExM in combination with Airyscan confocal imaging, which enables 3D super-resolution imaging of TCRs on the entire cell surface, has also shown significant accumulation of TCRs on microvilli (25). Visualization of microvilli on Jurkat T cells expressing a fluorescent protein-conjugated TCRζ by Airyscan confocal microscopy revealed high concentrations of TCRs, which specifically accumulated at the microvilli tips (**Figure 1D**) (46). Using LLSM, accumulation of endogenous TCRs and chimeric antigen receptors (CARs) on CAR- transduced human CD8^+^ T cell blasts were also observed in 50% and 87% of microvilli tips, respectively (54). However, in ovalbumin-specific TCR transgenic OT-I mice, TCR nano-clusters on CD8^+^ T cells were not restricted to microvilli, but increased microvilli occupancy was observed upon activation (55). Considering that microvilli are dynamic actin-based structures that undergo continuous transitions between growth, steady-state and retraction cycles (56), it is not surprising that some TCR clusters are not strictly localized in microvilli. It is also possible that the extent of TCR accumulation on microvilli may differ between human and mouse T cells or among different T cell subpopulations, and further studies may be needed to characterize these differences. Interestingly, the presence of high concentrations of B cell receptors (BCRs), which serve equivalent role to that of T cell TCRs, on the microvilli of human B cells has also been observed using super-resolution imaging (25). Although a couple of studies have reported a random distribution of TCRs on resting T cells (57, 58), the cells in these studies were spread on surfaces containing anti-CD45 antibodies or ICAM1, which can cause physical alterations or induce non-canonical signaling. This may explain the discrepancies between their results and other observations of pre-clustered TCRs. The reported features of TCR nanostructures studied in various cell types using different microscopic techniques are summarized in **Table 1**.

**Table 1:** TCRs on the quiescent state of T cell membrane.

Dimension	T cell type	Microscopic technique	Reported feature
2D	2B4 cells (40), CH7C17 cells (40), human peripheral blood T cells (PBTs) (40, 43, 49), mouse naive and memory CD4^+^ T cells (45), mouse CD3ζ-transduced-CD4^+^ T cells (47, 48), mouse naïve or resting T cells (47), Jurkat T cells (49), CD3ζ-transduced Jurkat T cells (50, 57), mouse CD4^+^ effector T cells (58)	Immunogold-EM (40, 45, 47), FCCS (47), PALM (47–49), dSTORM (48, 50, 58), TIRF-SIM (57), STED (58)	Oligomers (40, 45), protein islands (47, 48), nanoscale clusters (49, 50), randomly dispersed (57, 58)
3D	Human resting (24) and effector (24, 59) PBTs, human CD4^+^ PBTs (25), Jurkat T cells (59), CD3ζ-transduced-Jurkat T cell (46), mouse OT-I transgenic CD8^+^ naïve or blast cells (55, 60), CAR-transduced human or mouse CD8^+^ T cell (54), Lymph node-resident TCR-transgenic mouse T cells (51)	VA-TIRFM+dSTORM (24, 59), Airyscan (46), Airyscan+ExM (25), LLSM (54, 55, 60), Synaptic contact mapping (SCM) using Qdot (55, 60), dSTORM (51), SIM (51)	Enriched on microvilli (24, 25, 59, 60), enriched on microvilli tips (46), nano-patches independent of microvilli (55), nano-patches on microvilli tips (50%) (54), nano-CAR patches on microvilli tips (83%) (54), nanometer-scale protein islands (51)

It has been proposed that TCRs in nanoclusters are more efficient for TCR triggering because 80% of the phosphorylated TCRζ is present in clusters containing 30% of the total TCRζ (50). The mechanisms underlying the trafficking of TCRs to microvilli are largely unknown. Actin filaments or specific motor proteins may play a role in transporting or maintaining TCRs on microvilli, as there is evidence that destabilization of the actin cytoskeleton by latrunculin-A leads to dispersion of TCR pre-clusters located on microvilli (24). In addition, microvilli were reported to collapse upon chemokine-induced Rac1 activation (61). These findings also raise interesting questions, such as whether the degree or form of TCR pre-organization depends on the T cell type, whether it changes during T cell development or differentiation, and how it affects T cell immunity.

## CD45 pre-exclusion from the tips of microvilli

The tyrosine phosphatase CD45 is ubiquitously expressed in hematopoietic cells and has multiple isoforms produced by alternative splicing (62, 63). CD45 is known to play both positive and negative regulatory roles in T cell activation. CD45 can dephosphorylate both negative and positive regulatory tyrosine residues on lymphocyte-specific protein tyrosine kinase (Lck) (64–66), and CD45 depletion abolishes TCR-mediated signaling, supporting a positive effect on T cell activation (67–69). Other studies support a negative role of CD45, suggesting that its abundant phosphatase activity serves to prevent non-specific or undesirable activation of immune cells (64, 70). CD45 suppresses T cell hyperactivation in a wild-type context (64, 71), and CD45 directly inhibits TCR-induced phosphorylation of CD3 ITAMs, a hallmark of TCR activation (72). In addition, recent studies have reinforced the negative regulatory role of CD45 in inhibiting T cell activation in the absence of high affinity antigens (70, 73).

Upon activation, CD45 is observed to be largely excluded from the central region of the IS, where TCRs are highly accumulated (74, 75). This segregation of CD45 and TCRs during T cell activation has been proposed as a mechanism of TCR triggering (2). The phosphatase activity of TCR-proximal CD45 is negated by its physical separation from the IS, resulting in a shift in equilibrium to phosphorylation of the ITAMs on TCRs, and leading to T cell activation. Two major models have been proposed for the molecular mechanisms of CD45 exclusion. According to the kinetic segregation model, T cells form close contacts with APCs, which then drives small scale segregation of CD45 from the contact regions, resulting in initiation of TCR triggering (76, 77). In this model, the long extracellular domain of CD45 (~25-50 nm in length) causes its exclusion from the IS center due to the narrow gap (~13 nm) between the T cell and APC in this area, which is too short to accommodate the CD45 extracellular domain (77–79). Another model for CD45 exclusion proposes segregation by lipid rafts (80, 81). Approximately 95% of CD45 is found in the non-raft fraction of lipids, while a small fraction (<5%) of CD45 is detected in the raft fraction (80–85). Lipid raft crosslinking with cholera toxin B subunit (CTB) or TCR crosslinking have been shown to exclude CD45 from the aggregation of lipid rafts (81, 86, 87). Altered localization of CD45 in lipid rafts has been reported in patients with systemic lupus erythematosus (SLE) (88–90).

**Figure 2:**
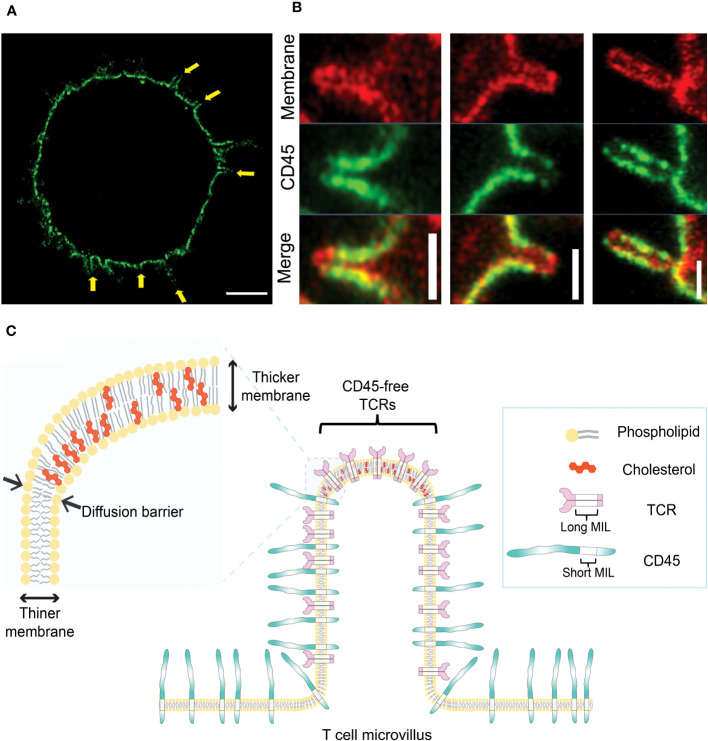
Pre-exclusion of CD45 from the tips of microvilli. **(A)** A 4x-ExM-Airyscan image a human resting T cell labeled with anti-CD45 antibodies (green) showing depletion of CD45 from microvilli tips (yellow arrows). **(B)** Magnified 4x-ExM-Airyscan images of microvilli on human CD4^+^ T cells labeled with anti-CD45 (green) and FM 4-64FX membrane dye (red), reproduced from Jung et al. (25), licensed under Creative Commons (CC BY 4.0). **(C)** Schematic illustrating a model of pre-exclusion of CD45 from the microvillus tip of T cell. High accumulation of cholesterol at the microvillus tip thickens the lipid bilayer at the microvillus tip, creating a diffusion barrier for CD45, which has a short membrane integration limit (MIL). Unlike CD45, TCR complex has long MILs, which allow TCRs to localize to the tip of microvilli; thus, TCRs localized at the microvilli tips are free from the phosphatase activity of neighboring CD45 (25).

Using ExM combined with Airyscan confocal microscopy and 3D STORM, we recently reported the novel organization of pre-formed of CD45 clusters in resting T cells, where CD45 is excluded from the tips of microvilli before contact with APCs (25) (**Figures 2A, B**). This feature of CD45 pre-exclusion from microvilli tips is common to human or mouse CD4^+^, CD8^+^, and regulatory T cells, as well as B cells. This CD45 pre-exclusion is distinct from the close contact-driven exclusion of CD45 from the microvilli tips reported by other groups (91, 92). We proposed that this pre-exclusion is caused by the diffusion barrier formed by the slightly thicker plasma membranes at the tips of microvilli. This is due to the local enrichment of cholesterol (lipid rafts), which blocks the free diffusion of CD45 whose transmembrane integration limit (MIL) is too short to integrate into the thicker membrane (**Figure 2C**) (25). We demonstrated that extraction of cholesterol using methyl-β-cyclodextrin (MβCD) significantly reduced the pre-exclusion of CD45 from microvilli tips. Mutant CD45 with increased MIL length displayed reduced pre-exclusion, whereas mutant CD45 lacking the extracellular domain maintained the pre-exclusion at the tips of microvilli, suggesting that CD45 pre-exclusion is mediated by the transmembrane domain of CD45 rather than the extracellular domain (25). This study supports the proposed lipid raft-driven CD45 mechanism, at least when T cells are in a quiescent state. The exclusion of CD45 from the tips of protrusions of unstimulated Jurkat T cells has also been reported by another group (93). In another recent study, we demonstrated that large-scale CD45 exclusion is directly induced by synthetic lipid rafts formed by DNA origami containing multiple cholesterols in the absence of ligation or close contact formation (94). This indicates that local cholesterol concentration plays a direct role in the formation of lipid rafts at the plasma membrane of live T cells as well as the exclusion of CD45 from them.

Given that the native form of the CD45 extracellular domain is physically excluded from residing at tight T cell-APC junctions and that TCR triggering induces the accumulation of lipid rafts at the interface, centric CD45 exclusion during IS formation is probably mediated by a combination of both mechanisms. The relative contribution of the extracellular CD45 domain *versus* the lipid raft-mediated CD45 exclusion during IS formation is not yet clear. However, our study (25) demonstrated that the pre-formed exclusion of CD45 from the tips of microvilli is directly mediated by the lipid composition of the membrane.

## Pre-clustering of coreceptors and proximal TCR signaling molecules

The coreceptors CD4 and CD8 bind to MHC-II and MHC-I class molecules, respectively. Other costimulatory receptors, CD2 and CD28, bind to LFA3 (CD58) and CD80 or CD86, respectively. These receptors play a key role in enhancing adhesion, sensitivity in antigen recognition, and T cell signal augmentation (95–99). Immunogold EM revealed that CD4 is concentrated in microclusters on microvilli of human T cells along with chemokine receptors CCR5 and CXCR4 (100). Roh et al. demonstrated that CD4 is localized in nanoclusters separated from the TCR or Lck at the resting state (48). Another study showed that CD4 fused to the photo-switchable protein mEos2 forms nanoclusters with an average diameter of 100 nm on the surface of resting Jurkat T cells, and that the CD4 nanoclusters are regulated by the extracellular domain and receptor palmitoylation (101). Another study using super-resolution optical fluctuation imaging (SOFI) (102) also reported high-density CD4 regions on the surface of Jurkat T cells (101). Haran’s group also demonstrated that more than 90% of CD4 and CD2 accumulate on the microvilli of human effector T cells and Jurkat T cells (59). In a 3D super-resolution imaging study, CD4 was also reported to accumulate at the microvilli tips, while CD45 was excluded from the tips in unstimulated Jurkat T cells (93). Cai et al. reported that CD8 on mouse OT-I T cells exists as pre-clusters located close to the TCR clusters before activation (55). They also reported that CD28 and the inhibitory receptor PD-1 (103) also form clusters, which are separated from TCR clusters in the quiescent state. These studies indicate that major coreceptors that modulate TCR-induced T cell activation are also not randomly distributed but, rather, exist as pre-clusters even before activation.

Similarly, TCR-proximal signaling molecules that propagate TCR triggering intracellularly, such as Lck kinase and the adaptor protein linker for activation of T cells (LAT) (104), are highly enriched on the microvilli of resting human effectors and Jurkat T cells (59). The microvilli-specific fractions of Lck and LAT were lower than that of TCR, CD4, and CD2, but still quite high at 76% and 68%, respectively. The size of these clusters ranges from 80 to 170 nm, which is similar to the diameter of a microvillus. Previously, Lillemeier et al. reported that LAT is found in clusters distinct from TCR clusters in the plasma membrane of T cells that were flattened on artificial surfaces in the absence of stimulation. In contrast, Sherman et al. reported pre-existing LAT nanoscale clusters that partially overlap with TCR clusters on T cells spread on non-stimulating surfaces (49). The authors also reported that in the absence of stimulation, LAT nanoclusters either partially co-cluster or not with ζ chain-associated protein kinase 70 (Zap70) (105) or with phospholipase C-gamma1 (PLC-γ1) clusters (106, 107), the latter two signaling molecules known to be recruited to the TCRs or LAT, respectively, upon TCR stimulation,. Interestingly, another study also reported that the adaptor protein SH2-domain-containing leukocyte protein of 76 kDa (SLP-76), a cytosolic adaptor known to be recruited to LAT upon T cell activation (108), is recruited to the rim of the LAT clusters on the activating artificial surfaces and, furthermore, that the nano-organization of LAT and SLP-76 is dependent on actin polymerization. These observations may reflect alterations of microvilli as they spread over a 2D surface. It remains unclear whether these signaling molecules are individually present in small clusters on microvilli, but these findings suggest that pre-organized proximal signaling molecules, as well as coreceptors required for early TCR signaling, may be co-localized on a single microvillus. Although the pre-cluster formation of LAT is known to be regulated by protein-protein or protein-lipid interactions (49), the molecular mechanism of the formation of these pre-formed coreceptors and signaling molecules in microclusters is mostly unknown. Together, the high accumulation of these coreceptors, costimulatory receptors, and proximal signaling molecules on microvilli allows for their physical proximity to the TCRs, which favors efficient transmission and amplification of early TCR signals.

## ERM proteins in microvilli

**Figure 3:**
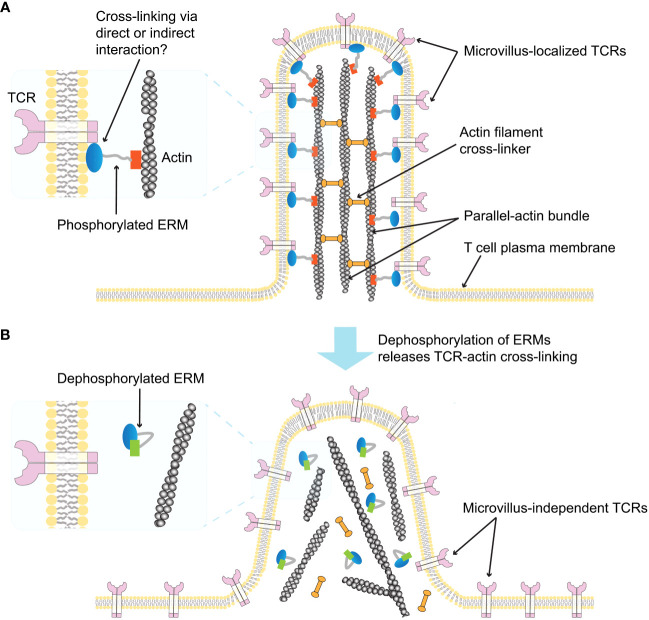
Schematic illustration of ERM-mediated TCR localization on microvilli (59). **(A)** A single T cell microvillus containing an actin bundle connected by actin-filament crosslinking proteins is depicted. The phosphorylated form of ERM crosslinks parallel actin filaments to the microvillus membrane, and the membrane-bound ERM links the TCR to the microvillus membrane (enlarged left). It is unclear whether the interaction between ERM proteins and the TCR is direct or indirect. **(B)** Dephosphorylation of ERM proteins releases TCR-actin crosslinks (enlarged left), abolishing the TCR confinement on the microvillus and inducing microvillus collapse.

The ERM proteins, ezrin, radixin, and moesin, which belong to the band 4.1 superfamily, play an important role in cross-linking the cytoskeleton and plasma membrane (109, 110). Ezrin was first discovered at the intestinal brush border and is known to be highly expressed in intestinal microvilli (111). Radixin was originally purified as a barbed end-capping actin regulatory protein from adherent junctions isolated from rat liver (112), while moesin was isolated as a from bovine uterus heparin-binding protein (113). These three proteins are highly homologous, and binding to actin filaments via their C-terminal domains is induced by their phosphorylation. Once phosphorylated, each ERM protein can cross-link the actin cytoskeleton to specific plasma membrane proteins via their conserved N-terminal FERM domain (110, 114, 115) (**Figure 3A**). Although ERM proteins have been reported to be concentrated in microvilli at the intestinal brush border (114, 115), their distribution in relation to T cell microvilli was revealed in a recent study using combinatory super-resolution microscopic techniques (59). This study showed high colocalization of phosphorylated ERM proteins with TCRs and actin filaments in microvilli, suggesting that ERM proteins may stabilize TCR assemblies within microvilli prior to TCR ligand binding and T cell activation (**Figure 3A**). It was also shown that activation of the TCRs induces dephosphorylation of ERM proteins within minutes, leading to rapid dissociation of the actin cytoskeleton from the plasma membrane (116) (**Figure 3B**). These results imply that the preassembly of ERM proteins may limit the release of microvilli-localized TCRs from the actin cytoskeleton prior to initial TCR activation. However, further studies are needed in order to determine whether the interaction between the TCR and ERM proteins is direct or not, and whether the collapse of ERM protein-stabilized microvilli by actin depolymerization upon T cell activation affects the positioning of TCRs on microvilli.

## Adhesion and chemokine receptors

T cells physically interact with their surrounding environment or other cells through a variety of adhesion molecules or chemokine receptors while migrating, homing, arresting, and rolling on the endothelium, or attaching to high endothelial venules in peripheral lymph nodes. Investigations of the localization of these proteins on 3D cell surfaces have relied heavily in the past on immunogold-EM. These studies have shown that L-selectin (117–119), integrins α4β7, α4β1, α5β1, α6Aβ1, αvβ3, and αEβ7 (120, 121), and the chemokine receptors CCR5 and CXCR4 (100) are preferentially localized to the microvilli tips of T cells, whereas CD44 (117) and integrins LFA-1 (CD11a/CD18 or αLβ2) (121, 122) and Mac-1 (CD11b/CD18 or αMβ2) (121) are mainly localized on the flat cell body. Modern super-resolution imaging techniques have also been applied to map the nanoscale distribution of these proteins in T cell plasma membrane. High accumulation of L-selectin (CD62L) on resting T cell microvilli has been demonstrated using several super-resolution imaging techniques (24, 25, 55). In contrast to L-selectin, CD44 has been reported to be localized on the cell body (117, 119). Lately, super-resolution imaging of resting effector human T cells has shown that CD44 accumulates around the base of the microvilli projections but not on the microvilli (24). Recently, the nanoscale topographical distribution of the chemokine receptor CCR7 (a member of G-protein coupled receptor family) and LFA1 has been reported. CCR7 clusters at the tips of microvilli, and 5% of LFA1 is found within a distance of ~20 nm from the CCR7 clusters, while the majority of the LFA1 is located on the cell body in the resting state (123). In addition, RhoA, a key GTPase involved in LFA-1 activation by CCR7-binding chemokines, is highly colocalized with CCR7 at the tips of microvilli. The tyrosine kinase JAK2, which is also involved in rapid CCR7-mediated LFA-1 activation, is also highly accumulated at the microvilli tips of human T cells (123). These results indicate that microvilli may function as signaling hubs ready to respond extremely rapidly to chemokine signals that play an important role in the arrest of T cells that roll on specialized vascular endothelial cells (124). Although it is unclear which membrane or cytoskeletal effector molecules control this exceptional segregation of chemokine receptors and adhesion receptors, the localization of pre-organized adhesive and signaling assemblies on or outside microvilli may also influence the mechanical forces experienced by T cells interacting with endothelial ligands under shear flow (125).

## Preassembly of Flot1-rich domain on T cells

Flotillin-1 (Flot1 or Reggie 2) and flotillin-2 (Flot2 or Reggie 1) are ubiquitously expressed scaffold proteins known to localize to lipid rafts in the of plasma membrane of mammalian cells (126–129). Flot1 and Flot2 are oligomerized and are linked to the inner leaflet of the plasma membrane via palmitoylation or myristylation, respectively (130, 131). Interestingly, in T cells, flotillins are known to form polarized cap-like preassembled clusters on the plasma membrane in the resting state (132, 133) and these micron-sized preassembled flotillins are highly stable (94, 133). This preassembly of flotillins is a common feature of hematopoietic cells (132). The accumulation of flotillins in pre-clustered caps is greatly enhanced by cross-linking of glycosylphosphatidylinositol (GPI)-anchored proteins (134, 135), cholera toxins (132, 135), or by anti-CD3/anti-CD28 antibodies (136), which recruit TCRs and TCR-proximal signaling proteins (*e.g*., Fyn, Lck, or LAT) to the flotillin-rich caps (132–136). In addition, chemokine stimulation causes a high accumulation of flotillin in the T cell uropod, where the adhesion molecule PSGL-1 and phosphorylated ERMs are localized to cause morphological polarization and induce cell motility (137).

The mechanism of flotillins preassembly is unclear. Although a correlation between cytokinesis and polarized flotillins has been reported (132), the molecular mechanisms underlying the formation of the polarized cap of flotillins remain elusive (132). Deletion of the flotillin C-terminus domain results in failure to form preassembled flotillin caps, and cotransfection with a mutant Flot2 lacking its N-terminal domain impairs preassembly formation of Flot2 (133). Although this preassembly defect does not affect the TCR-induced ERK1/2 kinase activation, Ca^2+^ signaling, or phosphorylation of Zap70, impaired assembly of Flot2 using the N-terminal deletion mutants caused changes in the intracellular localization of Vav, a guanine nucleotide exchange factor that affects cytoskeletal remodeling and cell spreading (133, 138).

The presence of this unique flotillin-rich micron-sized compartment in the plasma membrane of resting T cells is interesting as it is distinct from the mostly reported small-sized lipid rafts (~10-200 nm) present in the plasma membrane (14, 81). Unlike known lipid rafts, the stability of flotillin-rich microdomains is resistant to cholesterol depletion (132, 133). In a recent study, we utilized DNA origami constructs of cholesterol nano-patches (CNPs) to create synthetic lipid rafts on the resting T cell plasma membrane and found that the flotillin-rich domain highly colocalized with the synthetic lipid rafts (94). We also observed that CNP binding induces/enhances the polarization of Flot1. These results indicate that cholesterol can influence the formation and polarization of flotillin assemblies (94). We also found that polarized Flot1 was localized to the cell body and not to microvilli in Jurkat T cells (94).

Little is known about the function of flotillin in T cells. A recent study reported that flotillin is involved in sorting of TCRs during internalization upon anti-CD3/anti-CD28 costimulation, whereas TCR recycling back to the IS is flotillin-independent (139). Depletion of Flot2 demonstrated its essential role in CD44 expression, but its absence affects the cytoskeleton, polarity, and homing defects, as well as the progression of chronic myeloid leukemia (CML), while acute myeloid leukemia (AML) remains unaffected (140). Flot1 deletion in CD8^+^ T cells resulted in altered morphology and motility *in vivo*, but had only minor effects on clonal expansion, homing, and migration (141).

The cause(s) of flotillins preassembly on the plasma membrane of resting T cells and its physiological role remains to be elucidated. It is also unclear whether the preassembled flotillins cause the segregation of lipid domains in the plasma membrane or other membrane proteins, or whether this preexisting domain differs from traditional lipid rafts. Similarly, it remains to be determined whether and how preassembled flotillins recruit lipid rafts-associated proteins, GPI-linked proteins, or cholera toxin, as well as IS-forming components, such as TCR, Lck, LAT upon TCR stimulation (142). Further studies will hopefully resolve these questions and provide a better understanding of the role of preassembled Flot1-rich domains in T cell function.

## Implications and conclusions

**Figure 4:**
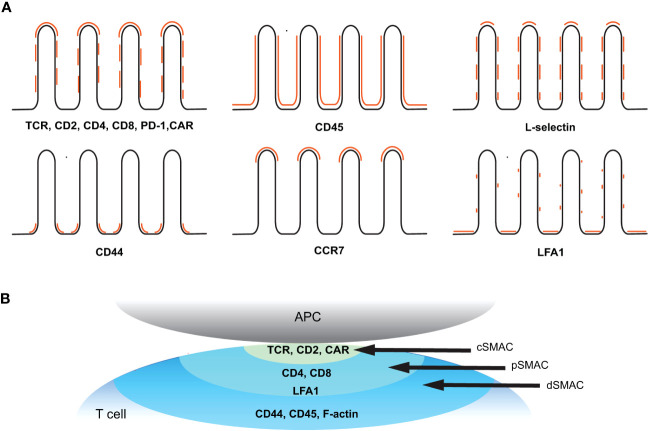
Spatial organization of key receptors before and after T cell activation. **(A)** Topographical distribution of key receptors on resting T cell membrane. Positions of the indicated receptors in relation to the microvilli (black line) are marked in orange. **(B)** Molecular distribution of key receptors within a classical immunological synapse after activation. The supramolecular activation cluster (SMAC) structure refers to the spatial organization of molecules within the IS. The central SMAC (cSMAC) contains TCRs and associated signaling molecules, the peripheral SMAC (pSMAC) surrounds the cSMAC and consists of costimulatory molecules and adhesion molecules, and the distal SMAC (dSMAC) at the outer edge of the synapse contains CD44, CD45, and F-actin.

Overall, many of the key receptors involved in T cell activation are not randomly distributed on the T cell plasma membrane but, rather, are present as nano-sized preclusters on the membrane prior to activation. In many cases, such as the TCR, Lck, LAT, CD4, or PD-1, the size of the cluster increases upon ligand binding and initiation of signal transduction (47–51, 55). In addition, significant fractions of many, if not all, of the pre-organized receptors are selectively associated with plasma membrane topography, specifically the microvilli structures, suggesting that microvilli play an important role in early T cell activation. The 3D topographical distribution of the major surface receptors or other proteins in relation to the microvilli structure discussed in this review is summarized in **Figure 4A** and **Table 2**. Upon activation, these molecules undergo active redistribution, and the dynamics and spatial arrangement of these receptors in IS formation are highly influenced by the strength and duration of antigenic stimulus, and the stage of activation. The structure of the IS has been described as classical synapses (centralized and symmetric configuration) (**Figure 4B**), multifocal synapses containing multiple foci of molecular clusters, and motility and asymmetric kinases, extensively reviewed elsewhere (143, 144). The spatial arrangement of receptors in fully developed classical synapses is depicted in **Figure 4B**. Nevertheless, there is still a lack of understanding how the three-dimensional molecular distribution of these elements evolves in the context of microvilli topographical alterations during the early stages.

**Table 2:** Receptors or proteins in relation to microvilli.

Microvilli-localized proteins	Cell body-specific proteins	Others
TCR (24, 25) [Tip (46, 54, 55)], CAR (54), CD4 (59, 93, 100), CD2 (59), Lck (59), LAT (59), PD1 (55), ezrin (59), radixin (59), moesin (59), L-selectin (24, 117, 118) [tip (25, 55)], integrins (α4β7, α4β1, α5β1, α6Aβ1, αvβ3, and αEβ7) (120, 121), CCR5 (100), CXCR4 (100), CCR7 (123), JAK2 (123), RhoA (123)	LFA1 (121–123), Mac-1 (121), flotillin [preassembled cap (94)]	CD44 [rim at the bottom of microvilli (24), or cell body (117)] CD45 [excluded from microvilli tips (25, 93)]

Because T cells use their highly dynamic microvilli to efficiently scan the opposing surface of APCs within minutes (60), it is likely that TCRs, coreceptors, costimulatory receptors, and adhesion receptors preassembled on microvilli are the first molecules interacting with their microenvironment, including antigens presented on APCs. Furthermore, the exclusion of CD45 from the tips of microvilli may allow TCRs to efficiently and very rapidly recognize antigen and initiate contact and triggering during the microvilli-mediated scanning process (25). This implies that microvilli can serve as effective sensors for antigen recognition and signaling hubs for early stage T cell activation by TCR as well as by GPCR signaling (12, 24, 145).

Our understanding of the underlying mechanisms and functional consequences of microvilli in T cell immunity has recently expanded (125, 146–149). It has been demonstrated that microvilli-mediated contacts induce initial calcium triggering (25, 150–152) and that stabilization of the microvilli-mediated close contacts affects effector functions (54, 152). A study using nonporous membranes with varying pore depths, sizes, and interpore distances showed that microvilli confined in 200-nm pores altered gene expression and segregation of signaling molecules that lead to T cell activation and proliferation (153). A theoretical model study suggested that the size and dwell time of TCRs in CD45-depleted T cell-APC close contact regions (likely the microvilli tips), which are constrained by surface topography, are determinants of TCR triggering and ligand discrimination (154). Another recent study demonstrated that microvilli on T cells breach the highly charged glycocalyx barrier on target cells by formation of multiple close contacts via CD2-CD58 interactions while excluding CD45, thereby enabling sensitive antigen discrimination (152). Computational simulation of microvilli dynamics showed that microvilli motility optimizes the balance between scanning speed and antigen sensitivity, promoting positive signaling outcomes while moderately affecting antigen recognition (155).

In addition, the interplay between mechano-transduction and plasma membrane topography has been proposed to play a regulatory role in T cell activation (125, 148, 156, 157). The thin and flexible nature of microvilli influences mechanical forces, such as bending, tension, traction, shear, or the direction of forces exerted on TCR-pMHC binding. A theoretical study suggested that mechanical forces generated by the active motion of scanning microvilli stabilize the catch-bond (stimulatory bond) between TCR and pMHC, resulting in microvilli immobilization to promote further receptor engagement (158). It has also been reported that actin-based protrusions on cytotoxic T cells enhance their killing potency (159) and that actin-rich invadosome-like protrusions on memory and effector T cells can more easily penetrate glycocalyx-coated cells, especially cells of mesenchymal origin (*e.g*., endothelial and epithelial cells), to facilitate antigen recognition and calcium influx (160).

The unveiling of pre-organized receptor configurations on the T cell plasma membrane has shed light on the conundrum of how resting T cells are ready to rapidly and effectively respond to antigenic challenges. Before being activated, T cells are equipped with these pre-organized receptors on their surface, giving them an advantage in initiating and modulating selective and sensitive responses. The mechanisms underlying the formation and maintenance of these pre-organized receptors are largely unknown, and their physiological functions remain to be further elucidated. A better understanding of the topographical distribution of key surface receptors and the mechanisms that pre-organized these surface receptors on the T cell plasma membrane will help elucidate how T cells generate highly selective and sensitive responses to rare signals (*e.g*., binding of very few peptide-MHC complexes to the TCR). This will also expand our understanding of subsequent T cell activation, and such knowledge will potentially be highly beneficial for designing T cell-based therapeutics and vaccines.

## Author contributions

YJ: Conceptualization, Data curation, Funding acquisition, Project administration, Writing – original draft, Writing – review & editing.
